# Trends, causes and solutions of maternal mortality in Jinan, China: the epidemiology of the MMR in 1991–2020

**DOI:** 10.1186/s12889-021-11816-3

**Published:** 2021-10-05

**Authors:** Dafang Yu, Lihua Zhang, Shimin Yang, Qing Chen, Zhongliang Li

**Affiliations:** 1 Department of Nursing, Jinan Maternity and Child Care Hospital Affiliated to Shandong First Medical University, Jinan, China; 2 Department of Medicine, Jinan Maternity and Child Care Hospital Affiliated to Shandong First Medical University, Jinan, China; 3 Department of Public Health, Jinan Maternity and Child Care Hospital Affiliated to Shandong First Medical University, Jinan, China; 4 Department of Human Resources, Jinan Maternity and Child Care Hospital Affiliated to Shandong First Medical University, Jinan, China; 5 Department of Women Healthcare, Jinan Maternity and Child Care Hospital Affiliated to Shandong First Medical University, Jinan, 250012 People’s Republic of China

**Keywords:** Maternal mortality ratio, Time trend, Causes, Solutions

## Abstract

**Background:**

China was one of the few countries to achieve the Millennium Development Goals 5. China had taken many effective measures to reduce maternal mortality ratio (MMR) and has achieved encouraging progress. These measures were worth sharing for other countries to reduce the MMR, but the introduction of these measures from the national perspective was too grand, and the measures implemented in a city and the results achieved were more valuable. However, there were few studies on the prevalence and trends of prolonged maternal mortality in a city. In this study, we mainly introduced the prevalence of the MMR in Jinan,China from 1991 to 2020, analyzed the causes of trends and put forward some solutions to the difficulty existing in the process of reducing the MMR,hoping to serve as a model for some developing cities to reduce MMR.

**Methods:**

We collected maternal mortality data from paper records, electronic files and network platforms. The time trend of MMR was tested by Cochran-Armitage Test (CAT). We divided the study period into three stages with 10 years as a stage and the Chi-square test or Fisher’s exact test was used to test the difference in MMR of different periods.

**Results:**

From 1991 to 2020, We counted 1,804,162 live births and 323 maternal deaths, and the MMR was 17.93 per 100,000 live births. The MMR declined from 44.06 per 100,000 live births in 1991 to 5.94 per 100,000 live births in 2020, with a total decline of 86.52% and an annual decline of 2.89%. The MMR declined by 88.54% in rural areas, with an average annual decline 2.95%, faster than that in urban areas (82.06, 2.73%). From 1991 to 2020, the top five causes of maternal deaths were obstetric haemorrhage (4.55 per 100,000 live births), amniotic fluid embolism (3.27 per 100,000 live births), pregnancy-induced hypertension (2.61 per 100,000 live births), heart disease (2.33 per 100,000 live births) and other medical complications (2.05 per 100,000 live births). Postpartum hemorrhage, amniotic fluid embolism, pregnancy-induced hypertension showed a downward trend (*P* < 0.05) and other medical complications showed an upward trend (*P* < 0.05).

**Conclusions:**

Subsidy for hospitalized delivery of rural women, free prenatal check-ups for pregnant women and rapid referral system between hospitals have contributed to reducing MMR in Jinan. However, it was still necessary to strengthen the treatment of obstetric hemorrhage by ensuring blood supply, reduce the MMR due to medical complications by improving the skills of obstetricians to deal with medical diseases, and reduce the MMR by strengthening the allocation of emergency equipment in county hospitals and the skills training of doctors.

## Introduction

The maternal mortality ratio (MMR) was not only an important indicator of maternal and infant safety, but also an indicator for judging a country’s or region’s economy, education and medical care [1]. Since reducing MMR became one of the eight Millennium Development Goals (MDGs) [2]. Countries around the world have paid more attention to it and have taken various measures to achieve it. The global MMR declined from 281.5 per 100,000 live births in 1990 to 195.7 per 100,000 live births in 2015 and the MMR dropped by 30.48%, an annual decline of 1.5% [3]. Although the MMR has been controlled, only 10 countries had achieved the MDG5, which required the MMR in 2015 be reduced by 75% compared with 1990. So countries around the world should make more efforts to reduce MMR, especially developing countries, because about 99% of maternal deaths occured in developing countries [4, 5] and many of them can be preventable [6–8].

The reasons for the slow progress on MDG 5 were varied. The AIDS epidemic [9], the gap between the rich and the poor [10, 11], the low status of women [12, 13] and other difficulties have prevented women from receiving adequate pregnancy or delivery care. In the process of reducing maternal deaths, China also faced similar difficulties. The Chinese government has taken a series of measures to overcome these difficulties. Acoording to the recommendations of WHO, China had implemented projects such as ‘Prevention of mother-to-child transmission of AIDS, syphilis and hepatitis B ‘and ‘Hospital delivery subsidy for Rural Women ‘. In addition, health education for pregnant women and wide promotion of obstetric technology for obstetricians in county hospitals also played an important role in reducing maternal mortality. The implementation of these measures had made China become one of the ten countries to realize MDG5 [3]. The national maternal mortality ratio in China declined from 114.2 per 100,000 livebirths in 1990 to 85.2 per 100,000 livebirths in 2000, and to 17.7 per 100,000 livebirths in 2015 [3].

Some studies have reported on strategies taken by China in the process of reducing MMR at the national, provincial and county levels [14–18]. However, there were few long-term studies on the long-term trend, measures taken and achievements achieved in a city. At the same time, in the process of shifting from MDG to SDG [19], China was facing a more complex environment, such as the adjustment of fertility policies [20], the increase of migrant women [21], and the low fertility desire of women [22]. These problems may also be faced in other developing countries.

Jinan is a central city in eastern China, with a total population of about 8.9 million, and about 80,000 new births every year. The MMR in Jinan declined from 33.89 per 100,000 livebirths in 1990 to 7.41 per 100,000 livebirths in 2015, and to 5.26 per 100,000 livebirths in 2020. Jinan’s goverment has made a lot of efforts to reduce MMR, including granting financial assistance rural women deliveried in hospital, providing free prenatal examination for pregnant women and establishing a green transit channel for critically ill pregnant women. Especially after the achievement of MDG5, In Jinan, all pregnant women were managed according to the severity of the disease, and hospitals and public health institutions paid close attention to the changes in the condition of pregnant women and implement timely and accurate interventions, which contributing to maintaining a downward trend in the MMR.

We select Jinan as the research site, analyzed the changes in the MMR from 1991 to 2020, and introduced detailedly the the implementation of policies and its achievements. Meanwhile, we also found some problems and proposed corresponding solutions. We believe that it can serve as a model for some developing cities to reduce MMR.

## Method

### Data collection

Data collection was based on the three-level network of the Maternal and Child Healthcare Institutions (MCH), including hospitals and village clinics, the county-level MCH and the city-level MCH. Hospitals and village clinics were the source of data collection, the county-level MCH was the hub of data collection, and the city-level MCH was the data collection, collation and analysis institution. If the maternal death occured in the hospital, the doctor will report the maternal death to county-level MCH. If the maternal death occurs outside the hospital, the village health worker also will report the maternal death to county-level MCH. The submitted data was verified by the county-level MCH and reported to the city-level MCH within 24 h. To ensure accuracy, the city-level MCH and the county-level MCH go to hospitals and household registration offices to conduct underreporting investigations of maternal deaths every 6 months.

From 1991 to 2000, data was recorded on paper and manually summarized, reported, and counted. From 2001 to 2011, the data was reported and counted through the “ National Maternal and Child Health Routine Reporting System (MCHRRS)”. Since 2012, Jinan’s goverment has designed the “Jinan Maternal and Child Health Information Platform”. This system covers all medical institutions, including hospitals, primary medical institutions and public health institutions. All pregnant women’s pregnancy, prenatal check-ups, childbirth, post-natal check-ups and other information were directly reported through the Internet. The data on maternal deaths after 2012 was more accurate and detailed because the data was aggregated on a case-by-case basis.

As to maternal deaths, the final determination of causes about maternal mortality had a very strict procedure. For maternal deaths inside the hospital, hospital records and other related information were collected by document reviewing or personal interviewing. For maternal deaths outside the hospital, information was collected by household visit. This sort of investigation focused on personal/family background, overall situation during pregnancy, onset of any disease and corresponding treatment, course of delivery, the date of termination of pregnancy, and the date of maternal death. Upon completion of the investigation, the investigation form, medical examiner reports, medical records, and autopsy reports for each case were submitted to the review committee at the county/city level, which consists of obstetricians, anaesthesiologists, pathologists, an emergency physician and some other specialists. A causal analysis report on each case of maternal death, including the most probable cause, identify preventable areas and suggest interventions was made by the Committee. Causes of maternal deaths were coded according to the ICD-10.

Tabulated data were provided on key maternal health metrics, including the time of delivery, the place of residence, the time of maternal deaths, the site of maternal deaths the cause of maternal death for all pregnant women. In the process of data collection, causes of maternal deaths were missing, especially some pregnant women who suddenly died in the road and home cannot be accurately confirmed due to the lack of sufficient medical records and we attributed it to “sudden death”. Fortunately, only 9 maternal deaths belong to this situation, which does not affect our research conclusions.

### Operational definitions

Maternal deaths were defined as the death of a woman while pregnant or within 42 days of pregnancy termination, irrespective of pregnancy duration or termination method, excluding deaths from intentional and unintentional injuries [23].

We used the standard maternal mortality ratio (MMR = maternal deaths/100,000 live births) to record maternal death over time.

We determined that the etiology as the fundamental cause, which refered to the disease or injury that caused a series of direct death events. In addition, some symptoms such as respiratory failure, hemorrhagic shock were not the cause of maternal death, leading to these symptoms of the primary disease can be the cause.

We define direct obstetric death as maternal death caused by obstetric complications in pregnancy (pregnancy, childbirth and puerperium) or medical operation intervention, negligence and improper handling, such as amniotic fluid embolism, ectopic pregnancy, pregnancy-induced hypertension, etc.

We define indirect obstetric death as maternal death caused by previous diseases or new diseases during pregnancy. Although these diseases are not caused by direct obstetric reasons, they are aggravated by the physiological effects of pregnancy, such as congenital heart disease, pulmonary tuberculosis, liver cirrhosis, etc.

### Statistical analysis

After the data were obtained from different sources, it was compiled with Excel2007, each variable was checked for completeness and consistency. After data was cleaned and coded, we used SPSS version 21 for Windows to analysis it. The trend in MMR was analyzed with Cochran-Armitage Test (CAT) including difference areas, difference maternal deaths causes, difference maternal deaths sites. We divided the study period into three stages with 10 years as a stage and the Chi-square test or Fisher’s exact test was used to test the difference in MMR of different periods. We used 2-tailed tests and a significance level of *P* < 0.05.

## Results

### **1. Trends of the MMR in Jinan from 1991 to 2020**

**Fig. 1:**
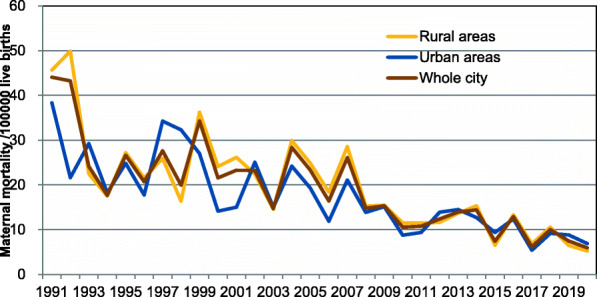
Trends of the MMR in Jinan from 1991 to 2020. Trends were analyzed by Cochran-Armitage trend (CAT) and showed significant reduction in both groups (the MMR in rural areas *P* *<* 0.0001, the MMR in urban areas *P* *<* 0.0001 and the MMR in whole city *P* *<* 0.0001). *Differences* were analyzed by *Chi-square test between the MMR in* rural areas and the MMR in urban areas and significant differences were observed

From 1991 to 2020, We counted 1,804,162 live births and 323 maternal deaths, and the MMR was 17.93 per 100,000 live births. The MMR was the highest in 1991 (44.06 per100,000 live births), and it was the lowest in 2020 (5.94 per 100,000 live births). The MMR in rural areas were statistically hihgher than that in urban areas (19.19 per 100,000 live births vs15.11 per 100,000 live births). The MMR decreased totally by 86.52%, an annual decrease of 2.89%, and the MMR declined by 88.54% in rural areas and 2.95% annually, faster than in urban areas (82.06, 2.73%) (Table 1, Fig. 1).

**Table 1:** The MMR in Urban and Rural Areas of Jinan during 1991–2020

year	Rural area*			Urban area*			Total*		
	Number of live birth	Number of maternal deaths	MMR	Number of live birth	Number of maternal deaths	MMR	Number of live birth	Number of maternal deaths	MMR
1991	37,231	17	45.66	10,431	4	38.35	47,662	21	44.06
1992	30,075	15	49.88	9254	2	21.61	39,329	17	43.23
1993	31,161	7	22.46	10,260	3	29.24	41,421	10	24.14
1994	34,267	6	17.51	10,970	2	18.23	45,237	8	17.68
1995	36,733	10	27.22	12,078	3	24.84	48,811	13	26.63
1996	41,888	9	21.49	11,261	2	17.76	53,149	11	20.70
1997	46,273	12	25.93	11,674	4	34.26	57,947	16	27.61
1998	42,769	7	16.37	12,385	4	32.30	55,154	11	19.94
1999	41,391	15	36.24	11,113	3	27.00	52,504	18	34.28
2000	41,451	10	24.12	14,167	2	14.12	55,618	12	21.58
2001	38,281	10	26.12	13,309	2	15.03	51,590	12	23.26
2002	40,070	9	22.46	15,966	4	25.05	56,036	13	23.20
2003	48,055	7	14.57	20,055	3	14.96	68,110	10	14.68
2004	43,439	13	29.93	16,523	4	24.21	59,962	17	28.35
2005	40,240	10	24.85	15,551	3	19.29	55,791	13	23.30
2006	38,059	7	18.39	16,829	2	11.88	54,888	9	16.40
2007	38,538	11	28.54	18,963	4	21.09	57,501	15	26.09
2008	39,330	6	15.26	21,644	3	13.86	60,974	9	14.76
2009	38,884	6	15.43	19,806	3	15.15	58,690	9	15.33
2010	43,377	5	11.53	22,829	2	8.76	66,206	7	10.57
2011	43,652	5	11.45	21,370	2	9.36	65,022	7	10.77
2012	43,001	5	11.63	21,620	3	13.88	64,621	8	12.38
2013	43,894	6	13.67	20,727	3	14.47	64,621	9	13.93
2014	45,703	7	15.32	23,641	3	12.69	69,344	10	14.42
2015	46,134	3	6.50	21,338	2	9.37	67,472	5	7.41
2016	45,053	6	13.32	32,342	4	12.37	77,395	10	12.92
2017	44,006	3	6.82	37,114	2	5.39	81,120	5	6.16
2018	47,370	5	10.56	32,760	3	9.16	80,130	8	9.98
2019	46,531	3	6.45	33,997	3	8.82	80,528	6	7.45
2020	38,271	2	5.23	29,058	2	6.88	67,329	4	5.94
total	1,235,127	237	19.19	569,035	86	15.11	1,804,162	323	17.93

Notes

*: Cochran-Armitage trend (CAT) *P* < 0.05

**Fig. 2:**
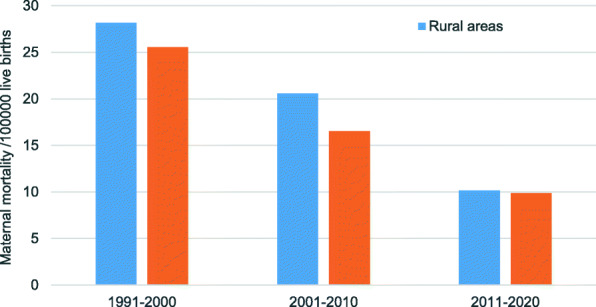
Differences in the MMR between rural and urban areas in different periods. Differences between rural and urban areas in different periods were analyzed by *Chi-square test*. Significant differences were observed in 1991–2000 (*P* = 0.003) and 2011–2010(*p = 0.005*), but not in 2011–2020 (*P* = 0.378)

From 1991 to 2000, the MMR in rural areas was 10.42% higher than that in urban areas (28.18 per 100,000 live births vs 25.52 per 100,000 live births). The gap was 24.44% from 2001 to 2010 (20.57 per 100,000 live births vs 16.53 per 100,000 live births) and 2.94% from 2011 to 2020(10.14 per 100,000 live births vs 9.85 per 100,000 live births). The gap of the MMR between rural areas and urban areas was decreasing (Fig. 2).

### **2. Trends of the MMR due to direct and indirect obstetric causes in Jinan from 1991 to 2020**

**Fig. 3:**
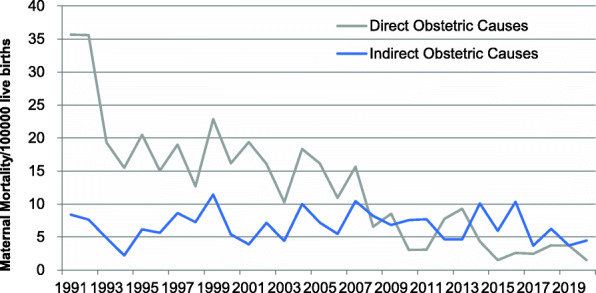
Trends of the MMR due to direct and indirect obstetric causes in Jinan from 1991 to 2020. Trends were analyzed by Cochran-Armitage trend (CAT) and showed significant reduction in both groups (direct obstetric causes *P* *<* 0.0001, indirect obstetric causes *P* < 0.0001*)*

From 1991 to 2020, 203 pregnant women (62.85%) died of direct obstetric causes and 120 pregnant women (37.15%) died of indirect obstetric causes. The MMR due to direct obstetric decreased by 95.82% in 2020 compared with 1991, with an annual decline of 3.19%. The MMR due to indirect obstetric decreased by 46.84% in 2020 compared with 1991, with an annual decline of 1.56% (Table 2, Fig. 3).

**Table 2:** The MMR Due to Direct and Indirect Obstetric Causes in Jinan during 1991–2020

Year	Direct Obstetric Causes*		Indirect Obstetric Causes*	
	n	MMR	n	MMR
1991	17	35.67	4	8.39
1992	14	35.60	3	7.63
1993	8	19.31	2	4.83
1994	7	15.47	1	2.21
1995	10	20.49	3	6.15
1996	8	15.05	3	5.64
1997	11	18.98	5	8.63
1998	7	12.69	4	7.25
1999	12	22.86	6	11.43
2000	9	16.18	3	5.39
2001	10	19.38	2	3.88
2002	9	16.06	4	7.14
2003	7	10.28	3	4.40
2004	11	18.34	6	10.01
2005	9	16.13	4	7.17
2006	6	10.93	3	5.47
2007	9	15.65	6	10.43
2008	4	6.56	5	8.20
2009	5	8.52	4	6.82
2010	2	3.02	5	7.55
2011	2	3.08	5	7.69
2012	5	7.74	3	4.64
2013	6	9.28	3	4.64
2014	3	4.33	7	10.09
2015	1	1.48	4	5.93
2016	2	2.58	8	10.34
2017	2	2.47	3	3.70
2018	3	3.74	5	6.24
2019	3	3.73	3	3.73
2020	1	1.49	3	4.46

Notes

n: Number of maternal deaths

MMR:Maternal Mortality Ratio (per 100,000 live births)

*: Cochran-Armitage trend (CAT) *P* < 0.05

**Fig. 4:**
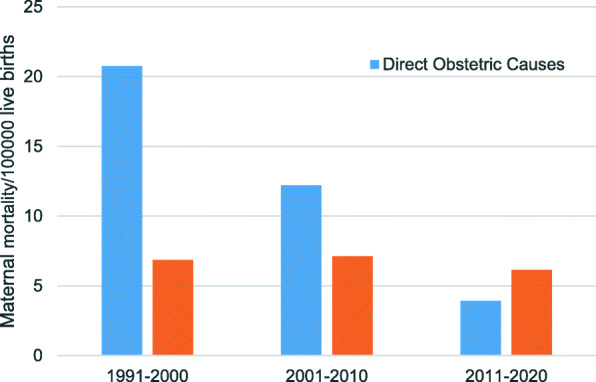
Differences of the MMR due to direct and indirect obstetric causes *in different periods. Differences* were analyzed by *Chi-square test* and significant differences were observed in 1991–2000 (*P* *= 0.001*),2011–2010(*P* *= 0.001*) and in 2011–2020 (*P* = 0.012)

We compared the MMR due to direct obstetric causes and indirect obstetric causes in different research periods. From 1991 to 2000, the MMR due to direct obstetric was 67.01% higher than that due to indirect obstetric (20.73 per 100,000 live births vs 6.84 per 100,000 live births). The gap was 41.68% from 2001 to 2010 (12.21 per 100,000 live births vs 7.12 per 100,000 live births). But, from 2011 to 2020, the MMR due to direct obstetric was 56.78% lower than that due to indirect obstetric (3.91 per 100,000 live births vs 6.13 per 100,000 live births) (Fig. 4). It mean that indirect obstetric causes had replaced direct obstetric causes as the main cause of maternal death.

### 3. **Trends of the MMR in top 5 causes of maternal deaths in Jinan from 1991 to 2020**

From 1991 to 2020, the top five causes of maternal deaths were obstetric haemorrhage (4.55 per 100,000 live births), amniotic fluid embolism (3.27 per 100,000 live births), pregnancy-induced hypertension (2.61 per 100,000 live births), heart disease (2.33 per 100,000 live births) and other medical complications (2.05 per 100,000 live births). Compared the MMR of the top 5 causes. Between 2020 and 1991, we found that in addition to other medical complications, the MMR due to the other four causes in 2020 was lower than that in 1990. Among them, obstetric hemorrhage and amniotic fluid embolism decreased by 100% with an annual decline of 3.33%, and pregnancy-induced hypertension decreased by 88.87% with an annual decline of 2.96%. In contrast, the MMR due to other medical complications increased by 100% in 2020 compared with 1991, with an annual increase of 3.33%. Trend test showed that heart disease had no significant upward or downward trend. (*P* > 0.05) (Table 3).

**Table 3:** The MMR due to Obstetric hemorrhage, Amniotic Fluid Embolism, Pregnancy-Induced Hypertension, Heart disease and Medical complications in Jinan from 1991 to 2020

Year	Obstetric hemorrhage*		Amniotic Fluid Embolism*		Pregnancy-Induced Hypertension*		Heart disease		Medical complications*	
	n	MMR	n	MMR	n	MMR	n	MMR	n	MMR
1991	7	14.69	3	6.29	6	12.59	1	2.10	0	0.00
1992	5	12.71	2	5.09	6	15.26	1	2.54	0	0.00
1993	4	9.66	2	4.83	1	2.41	2	4.83	1	2.41
1994	2	4.42	3	6.63	2	4.42	1	2.21	1	2.21
1995	4	8.19	5	10.24	1	2.05	1	2.05	0	0.00
1996	4	7.53	3	5.64	0	0.00	2	3.76	0	0.00
1997	4	6.90	3	5.18	3	5.18	1	1.73	1	1.73
1998	3	5.44	2	3.63	2	3.63	2	3.63	0	0.00
1999	7	13.33	5	9.52	0	0.00	2	3.81	1	1.90
2000	5	8.99	4	7.19	2	3.60	0	0.00	2	3.60
2001	3	5.82	5	9.69	3	5.82	1	1.94	2	3.88
2002	4	7.14	2	3.57	2	3.57	2	3.57	3	5.35
2003	3	4.40	1	1.47	2	2.94	0	0.00	3	4.40
2004	5	8.34	2	3.34	2	3.34	2	3.34	3	5.00
2005	2	3.58	2	3.58	3	5.38	3	5.38	3	5.38
2006	2	3.64	1	1.82	1	1.82	4	7.29	0	0.00
2007	1	1.74	3	5.22	1	1.74	1	1.74	2	3.48
2008	2	3.28	1	1.64	1	1.64	2	3.28	2	3.28
2009	3	5.11	1	1.70	0	0.00	1	1.70	0	0.00
2010	1	1.51	1	1.51	1	1.51	1	1.51	0	0.00
2011	1	1.54	0	0.00	0	0.00	2	3.08	1	1.54
2012	2	3.09	1	1.55	0	0.00	0	0.00	3	4.64
2013	3	4.64	1	1.55	1	1.55	2	3.09	0	0.00
2014	1	1.44	2	2.88	2	2.88	4	5.77	0	0.00
2015	1	1.48	0	0.00	1	1.48	1	1.48	1	1.48
2016	0	0.00	1	1.29	1	1.29	3	3.88	2	2.58
2017	1	1.23	0	0.00	1	1.23	0	0.00	1	1.23
2018	2	2.50	1	1.25	0	0.00	0	0.00	2	2.50
2019	0	0.00	2	2.48	1	1.24	0	0.00	1	1.24
2020	0	0.00	0	0.00	1	1.49	0	0.00	2	2.97
Total	82	4.55	59	3.27	47	2.61	42	2.33	37	2.05

Notes

n: Number of maternal deaths

MMR: Maternal Mortality Ratio (per 100,000 live births)

*: Cochran-Armitage trend (CAT) *P* < 0.05

**Fig. 5:**
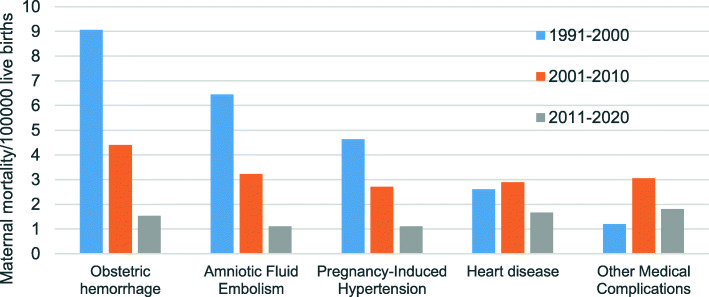
Differences in the MMR due to obstetric hemorrhage, amniotic fluid embolism, pregnancy-induced hypertension, heart disease and other medical complications *in different periods. Differences in MMR due to* obstetric hemorrhage, amniotic fluid embolism, pregnancy-induced hypertension, heart disease and other medical complications *in different periods* was analyzed with *Chi-square test*. The difference in obstetric hemorrhage, amniotic fluid embolism, gestational hypertension and other medical complications in different periods was statistically significant. (***P*** = 0.009, *P* = 0.012, ***P*** = 0.021, ***P*** = 0.007,) There was no significant difference of heart disease in different periods. (***P*** = 0.083)

We compared the MMR of the top 5 causes in different periods. Obstetric haemorrhage, amniotic fluid embolism and pregnancy-induced hypertension were gradually decreased. Among them, postpartum hemorrhage declined the most, which decreased by 83.09% in 2011–2020 compared with in 1991–2000. In contrast, other medical complications increased by 50.01% in 2011–2020 compared with in 1991–2000 (Fig. 5).

### 4. **Trends of the constituent ratio in sites of maternal deaths in Jinan from 1991 to 2020**

From 1991 to 2020, 136 pregnant women died in provincial hospitals (42.11%), 104 died in county hospitals (32.20%), 37 died in township hospitals (11.46%), 28 died in transport (8.67%) and 18 died in the home (5.57%).

The constituent ratio of maternal deaths in home, in transport and in township hospital decreased by 100% in 2020 compared with 1991, with an annual decline of 3.33%. The constituent ratio of maternal deaths in provinical/municipal hospital increased by 162.51% in 2020 compared with 1991, with an annual increase of 5.4%. The constituent ratio of maternal deaths in county hospitals had no significant upward or downward trend. (Table 4).

**Table 4:** The constituent ratio on sites of Maternal Deaths in Jinan from 1991 to 2020(%)

Year	Home*		In transport*		Township hospital*		County hospital		Provincial /municipal hospital*	
	n	%	n	%	n	%	n	%	n	%
1991	2	9.52	2	9.52	4	19.05	7	33.33	6	28.57
1992	1	5.88	2	11.76	5	29.41	5	29.41	4	23.53
1993	1	10.00	1	10.00	3	30.00	3	30.00	2	20.00
1994	0	0.00	1	12.50	3	37.50	2	25.00	2	25.00
1995	0	0.00	2	15.38	2	15.38	4	30.77	5	38.46
1996	0	0.00	3	27.27	1	9.09	3	27.27	4	36.36
1997	0	0.00	3	18.75	2	12.50	6	37.50	5	31.25
1998	0	0.00	0	0.00	2	18.18	4	36.36	5	45.45
1999	2	11.11	1	5.56	3	16.67	8	44.44	4	22.22
2000	1	8.33	1	8.33	1	8.33	6	50.00	3	25.00
2001	0	0.00	2	16.67	2	16.67	4	33.33	4	33.33
2002	2	15.38	1	7.69	1	7.69	4	30.77	5	38.46
2003	1	10.00	1	10.00	1	10.00	3	30.00	4	40.00
2004	1	5.88	2	11.76	3	17.65	5	29.41	6	35.29
2005	1	7.69	1	7.69	1	7.69	4	30.77	6	46.15
2006	1	11.11	1	11.11	1	11.11	3	33.33	3	33.33
2007	1	6.67	2	13.33	2	13.33	4	26.67	6	40.00
2008	0	0.00	1	11.11	0	0.00	3	33.33	5	55.56
2009	0	0.00	0	0.00	0	0.00	3	33.33	6	66.67
2010	1	14.29	0	0.00	0	0.00	2	28.57	4	57.14
2011	1	14.29	0	0.00	0	0.00	3	42.86	3	42.86
2012	0	0.00	0	0.00	0	0.00	2	25.00	6	75.00
2013	0	0.00	0	0.00	0	0.00	3	33.33	6	66.67
2014	1	10.00	1	10.00	0	0.00	3	30.00	5	50.00
2015	0	0.00	0	0.00	0	0.00	2	40.00	3	60.00
2016	0	10.00	0	0.00	0	0.00	2	20.00	8	80.00
2017	0	0.00	0	0.00	0	0.00	2	40.00	3	60.00
2018	0	0.00	0	0.00	0	0.00	2	25.00	6	75.00
2019	0	0.00	0	0.00	0	0.00	2	33.33	4	66.67
2020	0	0.00	0	0.00	0	0.00	1	25.00	3	75.00
Total	18	5.57	28	8.67	37	11.46	104	32.20	136	42.11

Notes: n: Number of maternal deaths

*: Cochran-Armitage trend (CAT) *P* < 0.05

**Fig. 6:**
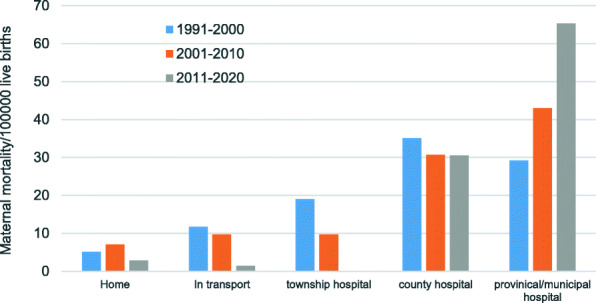
Differences in the constituent ratio of maternal deaths sites in different periods. In addition to the analysis of township hospitals in different periods using Fisher exact *test*, the analysis of other death sites using Chi-square test. The results showed that there were differences in home (*P* = 0.012), transport (*P* = 0.027), township (*P* = 0.001) and provinical/municipal hospital (*P* = 0.002), but not in county-level hospitals. (*P* = 0.091)

We also compared the constituent ratio of different maternal deaths sites in different periods. We found that the constituent ratio of maternal deaths in home, transport and town hospitals decreased significantly, especially after 2011, almost no maternal deaths occurred in these three places. On the contrary, the constituent ratio of maternal deaths died in municipal hospitals increased gradually, while the constituent ratio of maternal deaths died in county hospitals changed little (Fig. 6).

## Discussion

This study showed an overall downward trend in MMR in Jinan from 1991 to 2020, with an annualised rate of decline of 5.10% in 1991–2000, 5.46% in 2001–2010, 4.49% in 2011–2020 and 2.89% in 1991–2020. The annualised rate of decline in maternal mortality ratio in 1991–2020 was equivalent to High-middle SDI countries [3].

In 2015, the MMR in Jinan was 7.41 per 100,000 live births, which was lower than the national MMR (21.8 per 100,000 live births), but it was not one of the 20 districts with the lowest mortality rate in China [14]. Compared with other countries in the world, the MMR of Jinan was lower than that in the high-SDI countries (15.0 per 100,000 live births), which was equivalent to that of Belgium (7.4 per 100,000 live births) and Canada (7.3 per 100,000 live births), and higher than that of Singapore (5.0 per 100,000 live births), Finland (3.8 per 100,000 live births) and Italy (4.2 per 100,000 live births) [3]. By comparing with the MMR of other countries, we found, as an developing city, the achievement of reducing maternal mortality in Jinan was encouraging. The fllowing detailed measures can explain the reasons for the encouraging achievement.

Firstly, the reduction of maternal mortality in rural areas has contributed to it. Since 2009, Jinan’s government has implemented the “Rural Women’s Hospital Childbirth Subsidy Project”. Each rural woman who gave birth in the hospital can receive a subsidy of Ұ 500. In addition to the “ New Cooperative Medical Scheme” [24] implemented after 2007, the average cost for each rural woman to give birth in the hospital was less than Ұ 1000. The low cost of hospital delivery has greatly reduced the economic burden of rural women and increased the hospital delivery rate. The hospital delivery rate in Jinan rose from 78.23% in 1991 to 98.91% in 2012. After 2012, almost no pregnant women gave birth at home. Some studies have shown that 60% of maternal deaths occur after childbirth [25, 26]. Observing and giving birth to women in the hospital, which has adequate rescue equipment and skilled obstetricians [27], undoubtedly greatly reduces the risk of maternal deaths.

Secondly, it should be attributed to the continued implementation of the “Maternal Health Management Project”, which was one of 12 public health projects in Jinan. The project was free, including 5 prenatal check-ups, post-natal visits, health education, etc. Five prenatal examinations cover the entire pregnancy and childbirth period. The examination items include not only low-cost examinations such as height, weight, blood pressure and other examinations, but also high-cost examinations such as ultrasound, hemoglobin measurement, and liver function assessment. In 2020, the proportion of 5 prenatal check-ups in Jinan reached 86.79%. The measure realized the monitoring and timely intervention of the main vital signs of pregnant women, avoided more serious organ damage and prevented maternal deaths [28]. The significant reduction of MMR due to pregnancy-induced hypertension was the most direct effect of this measure.

Thirdly, a fast channel referral system for pregnant women also played an important role. Jinan’s government had implemented hierarchical management for all pregnant women since 2011. After pregnancy, all pregnant women go to township hospitals to establish files and undergo examinations. After examination, those elderly pregnant women, obese pregnant women and pregnant women with other diseases that may affect delivery must go to county hospitals for examination again. For pregnant women with asthma, severe anemia and other diseases, we rated as high risk, these pregnant women must receive prenatal examination and delivery in county hospitals; For pregnant women with heart disease, malignant tumors and other diseases, we rated as high risk, these pregnant women must undergo prenatal examination and delivery in municipal hospitals. The risk level of pregnant women was not fixed, and doctors need to constantly adjust it according to the changes of pregnant women’s condition. Doctors in township hospitals also need to contact pregnant women regularly to understand the health status of pregnant women and make medical guidance. This measure clarified the respective responsibilities of township hospitals, county-level hospitals, and city-level hospitals in ensuring the safety of pregnant women. If these hospitals failed to classify pregnant women in accordance with their duties, or even cause maternal deaths due to their work errors, they will be severely punished and even have their medical qualifications cancelled. That’s why almost no pregnant women died in home, in transport or in township hospitals after 2011.

In addition, the convenience of traffic roads saves the referral time of critically ill pregnant women between different hospitals and the progress of medicine improves the rescue ability of doctors in high-risk pregnant women, which played a role in reducing the MMR.

Although the maternal mortality rate in Jinan has been significantly controlled, with the deepening of urbanization, the increase of population mobility and the adjustment of fertility policy, further reducing maternal mortality still faces many challenges.

Firstly, We found that although the MMR due to obstetric haemorrhage showed a downward trend, obstetric haemorrhage remained the leading cause of maternal mortality. The conclusion was consistent with findings from previous studies, so the key to reducing maternal mortality was to reduce the incidence of obstetric haemorrhage [29]. Many studies have shown that maternal deaths due to obstetric haemorrhage can be prevented if awareness of vital sings, the initial treatment, the timing of maternal transport, and intra- or inter-hospital relations could be improved [30, 31]. So the skills of medical staff in early detection and intervention of obstetric haemorrhage need to be strengthened. Women’s health awareness should be improved through the implementation of government health education projects. The government also should introduce policies to give priority to guaranteeing the blood supply of obstetrics and gynecology in hospitals [32].

Secondly, We found that the proportion of maternal deaths in county-level hospitals reached 32.20% in 2020, and the trend did not show a significant downward trend. By analyzing these deaths, we found that maternal deaths in county hospitals were avoidable. With the general economic development and the improvement of road conditions, more and more rural pregnant women give birth to county hospitals. Our study showed that the MMR in rural areas was higher than that in urban areas, so county hospitals played a vital role in ensuring the safety of pregnant women. However, the county hospitals in Jinan had the same shortcomings as other county hospitals in China in terms of obstetric treatment capacity and medical equipment [33, 34]. So it was necessary to strengthen the training of obstetric disease treatment ability of doctors in county-level hospitals and epuip adequate medical equipment for county hospitals.

Thirdly, We found an upward trend in the MMR due to other medical complications, especially after 2015. Meanwhile, the trend of the MMR due to cardiovascular diseases did not show a significant downward trend. The MMR due to obstetric haemorrhage gradually decreased and the MMR due to other medical complications or cardiovascular diseases gradually increased, which was similar to that in the UK [35] and USA [36]. This trend was a result of the evolution of maternal mortality, that was, direct obstetric factors that were relatively easy to be interfered by obstetricians were under control, but medical factors that were more difficult for obstetricians to deal with were gradually emerging. Besides, the situation in Jinan was also affected by fertility policy. In 2016, Jinan implemented a comprehensive two-child policy. After the implementation of this policy the proportion of pregnant women over 35 years old reached 45.67%. It posed a severe challenge to reducing the MMR. Many studies have pointed out that the risk of other medical complications in elderly women was higher than that in other age groups [37–39]. The key measures to solve this problem was to strengthen the skills of obstetricians in dealing with internal diseases, and form a multidisciplinary emergency team within the hospital to deal with emergency pregnant women in time.

This study has its limitations. Firstly, because the data collection in the 1990s relied on manual work, the MMR may be underestimated, especially in rural areas where pregnant women died of abortion may not be reported. Secondly, maternal mortality is affected by many factors, such as economic development level, women’ s health awareness, and traffic conditions. Due to the lack of objective indicators to measure these factors, we were unable to distinguish clearly between the contributions of each individual factor on MMR. Thirdly, COVID − 19 had adverse effects on pregnant women. Although there was no maternal death due to COVID − 19 in Jinan in 2020, whether COVID − 19 aggravated the maternal death by influencing the mental health of pregnant women or other factors needed further study.

## Conclusion

Subsidy for hospitalized delivery of rural women, free prenatal check-ups for pregnant women and a rapid referral system between hospitals have contributed to reducing maternal mortality in Jinan. However, it is still necessary to strengthen the treatment of obstetric hemorrhage by ensuring blood supply, reduce the MMR due to medical complications by improving the skills of obstetricians to deal with medical diseases, and reduce the MMR by strengthening the allocation of emergency equipment in county hospitals and the skills training of doctors.

## Data Availability

The data-sets used and analysed during the current study available from the corresponding author on reasonable request.
